# Trial-Off-Catheter (TOC) protocol at Yishun community hospital

**DOI:** 10.1017/ash.2025.129

**Published:** 2025-09-03

**Authors:** Chng H.Y.P., Doctor S.D., Tan B.C.B., Lee K.K.

**Affiliations:** 1 Nursing Administration; 2 Medical Services

## Abstract

**Background/Aim:** Early removal of indwelling urinary catheters (IDC) is an effective strategy to prevent catheter- associated urinary tract infection (CAUTI). We hypothesized a standardized Trial-Off-Catheter (TOC) protocol would reduce catheter utilisation and CAUTI rates in a community hospital. We aim to reduce catheter utilisation ratio and CAUTI rate per 1000 catheter days by 10% over a period of three months (post-intervention) in three pilot wards with the highest catheter usage. **Methods:** An IDC review board and a TOC protocol were designed collectively as a team in Yishun Community Hospital based on literature reviews. Roadshows were conducted at the three pilot wards and implemented over 2 months. 2-weekly audit was conducted by Infection Control Nurses on 3 process measures, (1) IDC were reviewed appropriately with IDC review board, (2) Appropriate usage of TOC protocol, (3) Compliance with TOC protocol. **Results:** Within three months’ post-intervention, IDC utilisation ratio reduced by 18%, CAUTI rate per 1000 catheter days reduced by 45%, percentage of IDC reviewed appropriately increased to 91%, zero CAUTI event related to the use of protocol and 77% successful catheter removal with TOC protocol. **Conclusion:** CAUTI prevention require continuous effort with multimodal strategies and support from various stakeholders. A standardized TOC protocol can reduce variation in practices among physicians/ nurses with different levels of experience with TOC. A standardized care process ensures efficient utilization of resources to achieve the desired clinical outcomes for patients.

